# Proteomic Analysis of the Intestinal Resistance to Thyroid Hormone Mouse Model With Thyroid Hormone Receptor Alpha Mutations

**DOI:** 10.3389/fendo.2022.773516

**Published:** 2022-04-28

**Authors:** Yue Xi, Dan Zhang, Yue Liang, Zhongyan Shan, Xiaochun Teng, Weiping Teng

**Affiliations:** ^1^ Department of Endocrinology and Metabolism, Endocrine Institute, and Liaoning Provincial Key Laboratory of Endocrine Diseases, The First Hospital of China Medical University, Shenyang, China; ^2^ Department of Endocrinology and Metabolism, The Third Affiliated Hospital of Jinzhou Medical University, Jinzhou, China

**Keywords:** thyroid hormone receptor, intestine, proteomic analysis, bioinformatic, parallel reaction monitoring analysis

## Abstract

Thyroid hormone is critical during the development of vertebrates and affects the function of many organs and tissues, especially the intestine. Triiodothyronine (T_3_) is the active form and can bind to thyroid hormone nuclear receptors (TRs) to play a vital role in the development of vertebrates. The resistance to thyroid hormone α, as seen in patients, has been mimicked by the *Thra* ^E403X^ mutation. To investigate the mechanisms underlying the effect of TRα1 on intestinal development, the present study employed proteomic analysis to identify differentially expressed proteins (DEPs) in the distal ileum between homozygous *Thra* ^E403X/E403X^ and wild-type *Thra* ^+/+^ mice. A total of 1,189 DEPs were identified, including 603 upregulated and 586 downregulated proteins. Proteomic analysis revealed that the DEPs were highly enriched in the metabolic process, the developmental process, the transporter of the nutrients, and the intestinal immune system-related pathway. Of these DEPs, 20 proteins were validated by parallel reaction monitoring analysis. Our intestinal proteomic results provide promising candidates for future studies, as they suggest novel mechanisms by which TRα1 may influence intestinal development, such as the transport of intestinal nutrients and the establishment of innate and adaptive immune barriers of the intestine.

## Introduction

Thyroid hormone (TH) production is a tightly regulated process controlled by a classic negative feedback loop involving the hypothalamus, the pituitary gland, and the thyroid, which has led to the common name hypothalamus–pituitary–thyroid axis. Thyroxine (T_4_) and triiodothyronine (T_3_) are synthesized and secreted by thyroid gland follicular cells. T_3_, as the active metabolite of the thyroid hormone, is known to be important for the normal development and life of adult vertebrates, especially during the regulatory period of post-embryonic birth (1–3). T_3_ exerts its effects *via* thyroid hormone receptors (TRs), which are members of the nuclear hormone receptor protein superfamily (4, 5) and are known as T_3_-modulated transcription factors (6). T_3_ can activate or inhibit T_3_ target genes by regulating the activity of TRs (6, 7). In mammals, the diversity of TR proteins is encoded by the two genes *THRA* and *THRB* (6, 8) through the use of different promoters and/or alternative splicing (9, 10). There are four isoforms encoded by each of the TRα locus (TRα1, TRα2, TRΔα1, and TRΔα2) (11–13) and the TRβ locus (TRβ1, TRβ2, TRβ3, and TRΔβ3) (13–15).

In the past few decades, researchers have found that the intestine is one of the target organs of TH action (16, 17). First, TH was reported to be one of the essential regulators of gastrointestinal development during the progression of amphibian metamorphosis (18). Then, mouse studies reported that mutations in TRα resulted in intestinal defects, such as shorter villi, reduced apoptosis of the villi, reduced cell proliferation of the crypts, and decreased sucrase, lactase, and aminopeptidase enzymatic activities of the intestine (1, 19, 20). Recently, our group established a new TRα mutant mouse model (*Thra* ^E403X/E403X^ mouse), and we observed that the length of the small and large intestine was significantly shorter, the number of epithelial cells was reduced, and the length of the villi and the depth of the crypts were significantly decreased in 3-week-old *Thra* ^E403X/E403X^ mice compared to *Thra* ^+/+^ mice (21). These findings indicated that TRα1 plays an important role in intestinal development, which could be associated with the short lifespan and impaired postnatal development of *Thra* ^E403X/E403X^ mice.

In the present study, our aim was to further elucidate the molecular mechanisms underlying the effects of TRα1 on intestinal development. To this end, intestinal proteomics and bioinformatics analyses were adopted to identify differentially expressed proteins (DEPs) in the distal ileum between homozygous *Thra* ^E403X/E403X^ (Hom) and wild-type *Thra* ^+/+^ (Wt) mice.

## Materials and Methods

### Animal Maintenance and Sample Preparation

Three-week-old male Wt and Hom mice were used in our experiment, which were obtained by crossing heterozygous (*Thra* ^E403X/+^) mice. The mice were housed in a specific pathogen-free environment at 22 ± 2°C with an automatic 12-h light–dark cycle and free access to food and water. The mice were anesthetized with 1.2 ml/kg of 3% pentobarbital sodium by intraperitoneal injection, and the intestine was dissected along the Treitz ligament, separating the small intestine. The intestines were dissected longitudinally and washed thoroughly with 4°C precooled saline. One centimeter of the distal ileum was placed in a cryopreservation collection tube placed in dry ice during collection and then stored in liquid nitrogen until further protein extraction protocols. Five samples from each genotype (*Thra* ^E403X/E403X^ mice, *n* = 5; *Thra* ^+/+^ mice, *n* = 5) were utilized for the proteomic analysis. All animal experiments were approved by the animal experimentation ethics committee of the China Medical University.

### Protein Extraction and Digestion

The process of protein extraction and digestion was performed according to a previously published method (22). The samples were homogenized in liquid nitrogen and thoroughly ground into powder. Then, the cell powder was further homogenized by ultrasonication with lysis buffer containing 1% SDS and 1% protease inhibitor on ice. Then, the samples were gently mixed at room temperature, followed by centrifugation at 12,000 × *g* for 10 min at 4°C. The supernatant was transferred to a new tube, and the protein concentration was determined with the BCA protein assay kit (Thermo Fisher Scientific, Rockford, IL, USA) according to the manufacturer’s instructions and measured three times with a Multiscan Ascent photometer (Thermo Fisher Scientific, Rockford, IL, USA) at a wavelength of 570 nm.

For protein digestion, equal amounts of sample protein were used, and precooled acetone was used to precipitate the proteins. Then, the proteins were centrifuged at 4,500 × *g* for 15 min, and the supernatant was discarded and washed twice with cooling acetone. Then, the protein solution was reduced with 5 mM dithiothreitol for 30 min at 56°C and alkylated with 11 mM iodoacetamide for 15 min at room temperature in darkness. The protein solution was diluted by adding 200 mM TEAB. After ultrasonication by the sonicator, trypsin (Thermo Scientific, USA) was used at a 1:50 trypsin-to-protein mass ratio for digestion overnight at 37°C and a 1:100 trypsin-to-protein mass ratio for a second 4-h digestion.

### TMT Labeling and HPLC Fractionation

The peptides were desalted using a Strata X C18 SPE column (Phenomenex, USA) and vacuum freeze-dried. The peptides were dissolved in 0.5 M TEAB and labeled with the TMT kit/iTRAQ kit (Thermo Fisher Scientific) according to the manufacturer’s instructions. Then, the TMT/iTRAQ reagent was reconstituted in acetonitrile, the peptides were mixed and incubated for 2 h, and the samples were pooled and vacuum freeze-dried. The peptides were eluted using high-pH reverse-phase HPLC using a Thermo Betasil C18 column (particle size, 5.0 μm; 250 mm × 10.0 mm i.d.; Thermo Fisher Scientific, Waltham, MA, USA). Briefly, the peptides were combined into six fractions and dried by vacuum centrifugation.

### LC–MS/MS Analysis

High-resolution mass spectrometry was performed using a Q-exactive mass spectrometer coupled with an EASY-nLC 1000 liquid chromatograph instrument (Thermo Fisher Scientific). The column was inserted in a HPLC Accela instrument (constituted by an autosampler and a 600 pump) and coupled to a Q-exactive mass spectrometer (Thermo Fisher Scientific) for high-resolution mass spectrometry. Mobile phase A was 0.1% formic acid + 2% acetonitrile in water. Mobile phase B was 0.1% formic acid + 2% acetonitrile in water. A gradient of 40 min was run with 9 to 26% solvent B, 40–54 min was run with 26 to 35% solvent B, 54–57 min was run with 35–80% solvent B, and 57-60 min was run with 80% solvent B with a flow rate of 500.00 nl/min. After separation by HPLC, the peptides were ionized by nanospray ionization (NSI) and analyzed by a Q-exactive instrument (Thermo Fisher Scientific). Mass spectrometric detection was carried out on a Q Exactive Orbitrap MS (Thermo Fisher Scientific) equipped with an NSI source operated in positive-ion mode. The spray voltage parameter was set to 2.2 Kv, and the MS data were acquired at the following parameters: MS1 spectra were collected in the range of 400 to 1,500 *m*/*z* at 70,000 resolution. MS2 spectra were collected at a fixed 100 *m*/*z* at 35,000 resolution. For data-dependent acquisition-MS, the top 20 precursor ions were selected for fragmentation with stepped normalized collision energies of 28%. With an automatic gain control (AGC) target of 5 × 10E4 and a maximum injection time of 50 ms, the dynamic exclusion time was set to 30 s.

### Database Search and Quantitative Calculation of Protein

The MS/MS data were recorded and analyzed using Maxquant software, version 1.5.2.8 (Thermo Fisher Scientific). The obtained peptide sequences were searched against the UniProt Mus_musculus_10090 (17,032 sequences) database concatenated with a reverse decoy database. The false discovery rate was adjusted to <0.01.

The raw LC–MS datasets were first searched against the database and converted into matrices containing the reporter intensity of peptides across samples. The relative quantitative value of each protein was then calculated based on these intensity values by the following steps: first, the intensities of peptides (*I*) across all samples were centralized and transformed into their values of relative quantification (*U*) in each sample. The formula is as follows: *i* denotes the sample, and *j* denotes the peptide. *Uij* = *Iij*/mean (*Ij*); second, to adjust the systematic bias of the identified peptide amount among different samples in the process of mass spectrometry detection, the relative quantitative value of the peptide needed to be corrected by the median value as follows: NR*ij* = U*ij*/median (*Ui*); and third, the relative quantitative value of a protein (R) was calculated by the intensity median of its corresponding unique peptides. The formula is listed as follows: *Rik* = median (NR*ij*, *j*∈*k*), where *k* denotes the protein and *j* denotes the unique peptides belonging to the protein.

### Bioinformatic Analysis

Fold change >1.30 or <0.77 and *p <*0.05 were set as the significant thresholds for the DEPs. The DEPs were annotated into three categories based on gene ontology (GO) terms, including biological processes, cellular components, and molecular functions.

The protein pathway was annotated *via* the Kyoto Encyclopedia of Genes and Genomes (KEGG) database. Enrichment analysis was conducted for the KEGG pathway.

### Parallel Reaction Monitoring Analysis

To verify the results of the proteomics analyses, 20 DEPs were selected and measured by parallel reaction monitoring analysis (PRM). PRM is a targeted proteomics technology based on high-resolution and high-precision mass spectrometry, which can selectively detect target proteins and target peptides, thus achieving the absolute quantification of target proteins/peptides (23). The protein isolation and trypsinization procedures were conducted as detailed above. The tryptic peptides were dissolved in 0.1% formic acid (solvent A) and directly loaded onto a homemade reversed-phase analytical column. The gradient was comprised of an increase from 6 to 23% solvent B (0.1% formic acid in 98% acetonitrile) over 38 min, 23 to 35% in 14 min, climbing to 80% in 4 min, and then holding at 80% for the last 4 min, all at a constant flow rate of 700 nl/min on an EASY-nLC 1000 UPLC system. The peptides were subjected to an NSI source, followed by tandem mass spectrometry (MS/MS) in Q ExactiveTM Plus (Thermo) coupled online to the UPLC. The electrospray voltage applied was 2.0 kV. The *m*/*z* scan range was 350 to 1,000 for full scan, and intact peptides were detected in the Orbitrap at a resolution of 35,000. Peptides were then selected for MS/MS using NCE setting 27, and the fragments were detected in the Orbitrap at a resolution of 17,500. We used a data-independent procedure that alternated between 1 MS scan and 20 MS/MS scans. AGC was set at 3E6 for full MS and 1E5 for MS/MS. The maximum IT was set at 20 ms for full MS and auto for MS/MS. The isolation window for MS/MS was set at 2.0 *m*/*z*.

The PRM data were processed using Skyline (v.3.6). For the peptide settings, trypsin [KR/P] was used as the enzyme, and the maximum missed cleavage was set as 2. The peptide length was set as 8–25, variable modification was set as carbamidomethyl on Cys and oxidation on Met, and the maximum variable modification was set as 3. The transition settings were as follows: precursor charges were set as 2 and 3, ion charges were set as 1 and 2, and ion types were set as b, y, and p. The product ions were set from ion 3 to the last ion; the ion match tolerance was set as 0.02 Da.

### Statistical Analysis

After filtration, data were subjected to univariate and multivariate analysis to calculate the fold changes between the *Thra* ^+/+^ and *Thra* ^E403X/E403X^ mice samples. Statistical significance was determined using Fisher’s exact test with Benjamini–Hochberg’s corrected *P*-value <0.05. Hierarchical clustering analysis was conducted for the DEPs, based on the significant enrichments, using the “heatmap.2” function from the “gplots” R package. The proteomic analysis in our research was supported by Jingjie PTM BioLabs (Hangzhou, China).

## Results

### Quantitative Proteins

**Figure 1:**
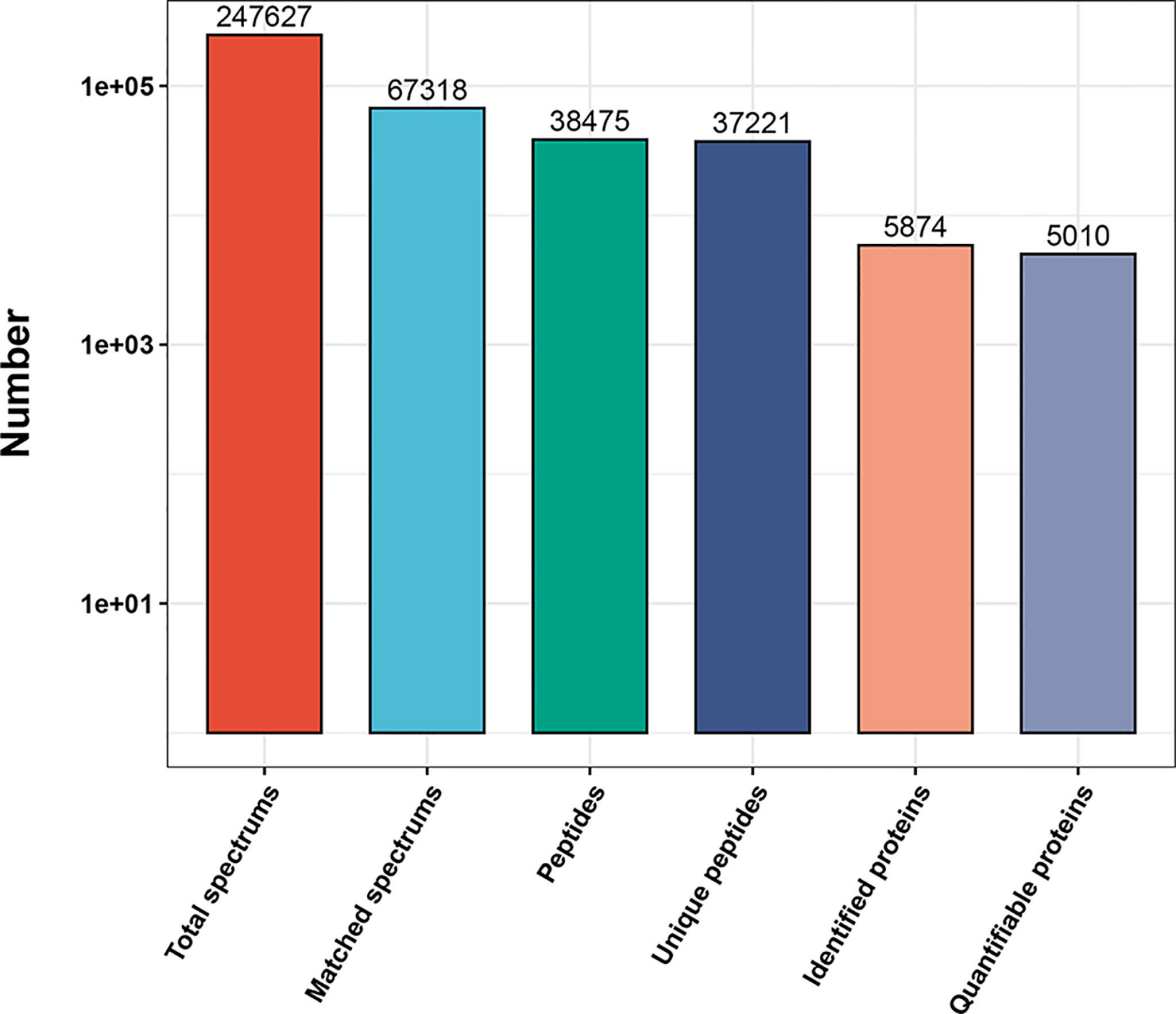
Overview of the mass spectrometry results. Bar plot summarizing the detected peptides and proteins in Wt *Thra* ^+/+^ (*n* = 4) and Hom *Thra* ^E403X/E403X^ (*n* = 5) mice.

Five mice from each genotype were initially selected (*Thra* ^E403X/E403X^ mice, *n* = 5; *Thra* ^+/+^ mice, *n* = 5). However, the biological repeat analysis showed that one Wt mouse was not reproducible with the other samples in the Wt genotype group, so the data from this sample were removed from the Wt group, leaving four biological replicates in the wild genotype and five biological replicates in the Hom genotype group for proteomic analysis. In total, 247,627 secondary spectrograms were obtained by mass spectrometry analysis. The number of available effective spectrograms was 67,318, and the utilization rate of the spectrograms was 27.2%. A total of 38,475 peptides were identified by spectrogram analysis, among which 37,221 were specific peptide segments. A total of 5,874 proteins were identified, of which 5,010 were quantifiable (**Figure 1**).

### DEP Analysis

**Figure 2:**
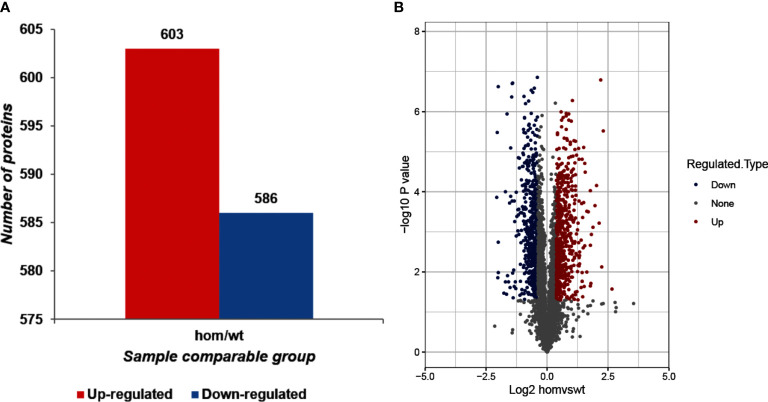
Identification of differentially expressed proteins (DEPs) between *Thra* ^E403X/E403X^ and *Thra* ^+/+^ mice. **(A)** The total number of upregulated and downregulated DEPs. The fold change (FC) >1.30 and *p*-value <0.05 were set as the significant thresholds for the upregulated DEPs. FC <0.77 and *p*-value <0.05 were set as the significant thresholds for the downregulated DEPs. **(B)** Volcano plot of the identified DEPs between *Thra* ^E403X/E403X^ (*n* = 5) and *Thra* ^+/+^ (*n* = 4) mice. The x-axis shows the log2 fold change of each protein, and the y-axis shows the *p*-value of the significant difference test after log10 logarithmic conversion. The red dots denote upregulated DEPs, the blue dots denote downregulated DEPs, and the gray dots denote unchanged proteins.

As shown in **Figure 2**, compared to *Thra* ^+/+^mice, 1,189 DEPs were identified in the *Thra* ^E403X/E403X^ mice, including 603 upregulated proteins and 586 downregulated proteins.

### Functional Enrichment Analysis of the DEPs

**Figure 3:**
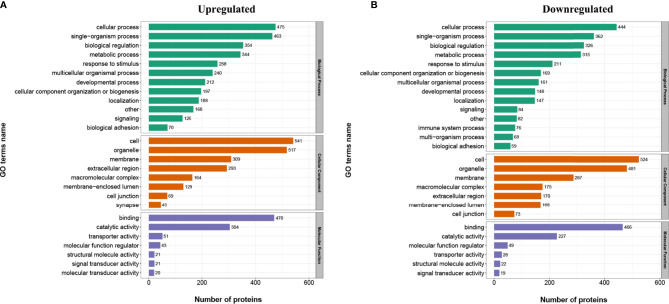
Gene ontology (GO) analysis for the identified differentially expressed proteins (DEPs). The DEPs were annotated into three categories based on GO terms, including biological processes, cellular components, and molecular functions. **(A)** GO enrichment analysis of the upregulated DEPs. **(B)** GO enrichment analysis of the downregulated DEPs.

**Figure 4:**
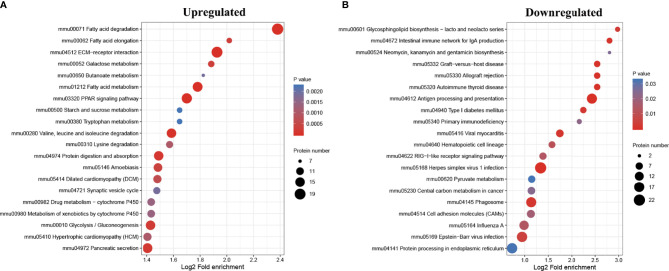
Kyoto Encyclopedia of Genes and Genomes (KEGG) pathway enrichment analysis of the differentially expressed proteins (DEPs). Bubble diagrams displaying the top 20 KEGG pathways for which upregulated **(A)** and downregulated **(B)** DEPs were significantly enriched, respectively. The y-axis shows the IDs and the names of the enriched pathways, and the x-axis shows the converted log2 fold enrichment. The size of the bubbles indicates the number of DEPs in each pathway.

In order to find out whether the DEPs have a significant enrichment trend in specific functional categories, we next conducted functional enrichment analysis of the DEPs based upon the gene ontology (GO) and Kyoto Encyclopedia of Genes and Genomes (KEGG) reference databases. Notably, the DEPs were significantly enriched for several relevant GO terms, including metabolic process, developmental process, and transporter activity (**Figure 3**). The top 20 enriched KEGG pathways are shown in **Figure 4**. Among them, several nutrient metabolism-related pathways were enriched, such as fatty acid degradation (mmu00071), fatty acid metabolism (mmu0121), fat digestion and absorption (mmu04975), starch and sucrose metabolism (mmu00500), and protein digestion and absorption (mmu04974). Furthermore, the antigen processing and presentation (mmu04612) and intestinal immune network for IgA production (mmu04672) pathways were enriched, and most of the DEPs related to these two pathways were downregulated.

### Hierarchical Cluster Analysis

**Figure 5:**
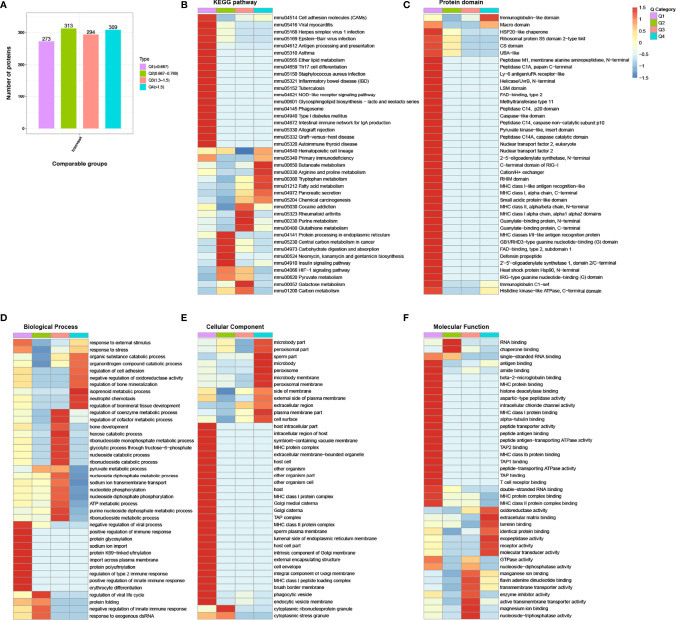
Hierarchical cluster analysis for the differentially expressed proteins (DEPs). **(A)** Fold change >1.30 or <0.77 and *p <*0.05 were set as the significant thresholds for the DEPs. According to the degree of fold change (FC), the DEPs were divided into four groups from Q1 to Q4. The different colors indicate the different Q categories: Q1 (FC ≤0.667, severely downregulated), Q2 (0.667 < FC ≤0.769, mildly downregulated), Q3 (1.3 < FC ≤1.5, mildly upregulated), and Q4 (FC>1.5, severely upregulated). **(B)** The distribution of Q categories in the Kyoto Encyclopedia of Genes and Genomes pathways. **(C)** The distribution of Q categories for the analysis of the protein domain. **(D)** The distribution of Q categories for the analysis of the biological process. **(E)** The distribution of Q categories for the analysis of the cellular component. **(F)** The distribution of Q categories for the analysis of the molecular function. The red color indicates stronger enrichment. The blue color indicates weaker enrichment.

According to the degree of the fold change, the DEPs were divided into four groups: severely downregulated (Q1; FC ≤0.667), mildly downregulated (Q2; 0.667 < FC ≤0.769), mildly upregulated (Q3; 1.3 < FC ≤1.5), and severely upregulated (Q4; FC>1.5) (**Figure 5A**). Some severely downregulated DEPs were enriched for the antigen processing and presentation and intestinal immune network for IgA production pathways (**Figure 5B**). In the protein domain cluster analysis, some severely downregulated DEPs were enriched for major histocompatibility complex class II (MHC class II), major histocompatibility complex class I (MHC class I), and caspase-like domain (**Figure 5C**). In the GO cluster analysis, some severely downregulated DEPs were enriched for MHC class II, MHC class I, and immune response GO terms (**Figures 5D–F**).

### PRM Validation

**Figure 6:**
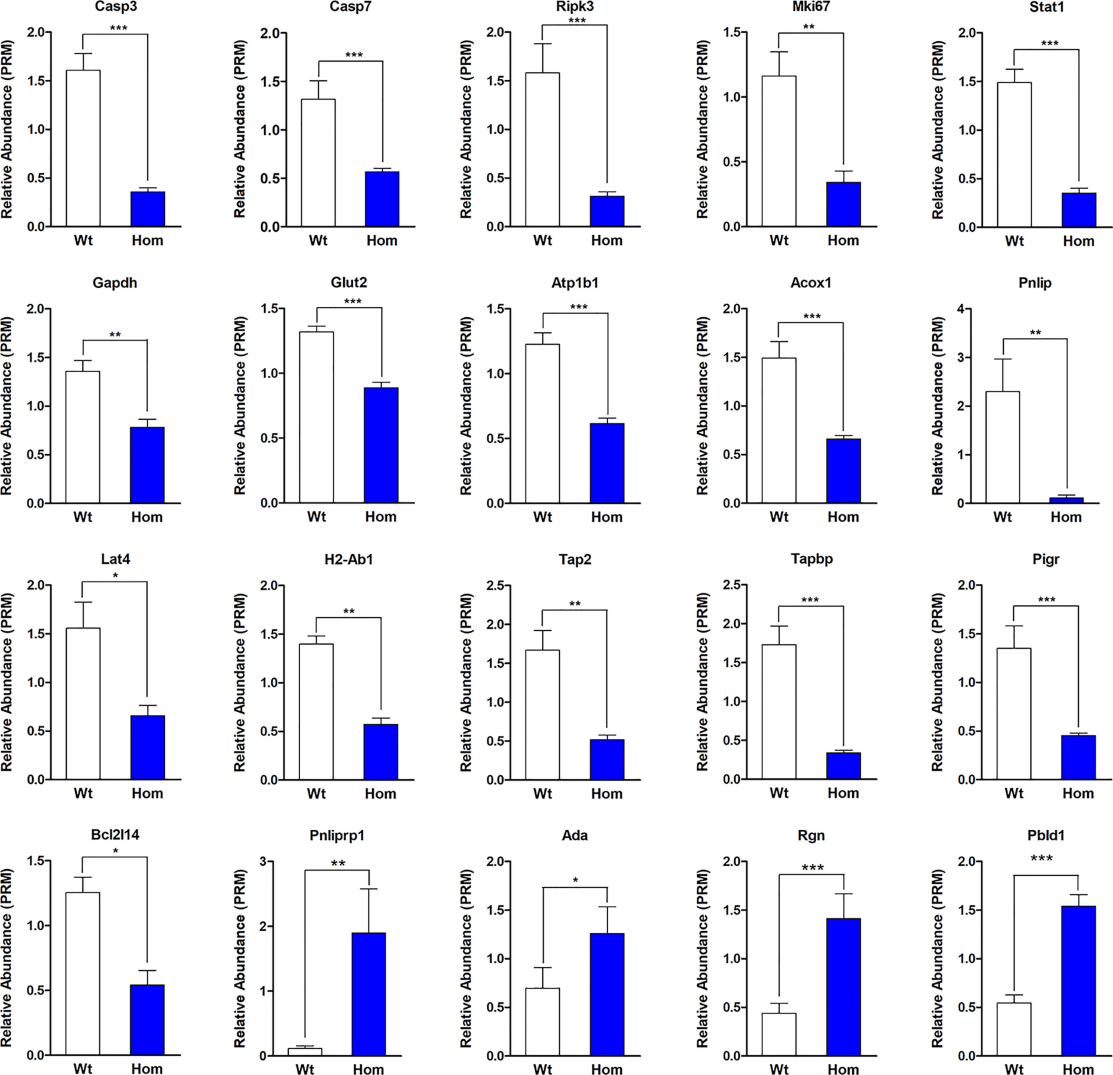
Validation by parallel reaction monitoring analysis. The absolute quantification levels of 20 differentially expressed proteins were evaluated in *Thra* ^+/+^ (*n* = 4) and *Thra* ^E403X/E403X^ mice (*n* = 5). Data are presented as mean ± SEM. Statistical analysis was performed by Student’s *t*-test. *P < 0.05; **P < 0.01; ***P < 0.001.

In order to verify our proteomic results, the protein levels of some genes reported to be regulated by T_3_ and the proteins that were enriched for GO terms or KEGG pathways were selected for parallel reaction monitoring (PRM) analysis. Validation by PRM analysis revealed that 20 proteins were significantly dysregulated in the Hom mice compared to Wt mice (**Table 1**). These proteins included caspase-3 (Casp3), caspase-7 (Casp7), and receptor-interacting serine/threonine-protein kinase 3 (Ripk3) for the apoptosis; proliferation marker protein Ki-67 (Mki67), regucalcin (Rgn), and adenosine deaminase (Ada) for cell proliferation; glyceraldehyde-3-phosphate dehydrogenase (Gapdh), glucose transporter type 2, liver (Glut-2), sodium/potassium-transporting ATPase subunit beta-1 (Atp1b1), inactive pancreatic lipase-related protein 1 (Pnliprp1), peroxisomal acyl-coenzyme A oxidase 1 (Acox1), pancreatic triacylglycerol lipase (Pnlip), and large neutral amino acid transporter small subunit 4 (Lat4) for nutrient metabolism; H-2 class II histocompatibility antigen, A beta chain (H2-Ab1), antigen peptide transporter 2 (Tap2), and Tapasin (Tapbp) for antigen processing and presentation; and polymeric immunoglobulin receptor (Pigr) for the intestinal immune network for IgA production. The PRM results showed that these candidate DEPs exhibited similar trends to those observed in the proteomic analysis, confirming the reliability of the proteomic data (**Figure 6** and **Table 1**).

**Table 1:** Parallel reaction monitoring (PRM) analysis of 20 candidate proteins.

Protein ID	Protein name	Gene name	Peptide sequence	Hom/Wt ratio (PRM)	Hom/Wt ratio (TMT)	Regulated type
P97864	Caspase-7	*Casp7*	VPTYLYR DLTAHFR	0.43	0.66	Down
Q9CPT0	Apoptosis facilitator Bcl-2-like protein 14	*Bcl2l14*	AQGPQGPFPVER TITDLFLR	0.43	0.65	Down
P70677	Caspase-3 OS=Mus musculus	*Casp3*	SVDSGIYLDSSYK SGTDVDAANLR	0.22	0.36	Down
Q9QZL0	Receptor-interacting serine/threonine-protein kinase 3	*Ripk3*	LHLEEPSGPVPGK GTTPGPVFTETPGPHPQR	0.20	0.51	Down
E9PVX6	Proliferation marker protein Ki-67	*Mki67*	SSGSTPVTAASSPK LPSSSPPLEPTDTSVTSR	0.29	0.76	Down
P42225	Signal transducer and activator of transcription 1	*Stat1*	DQQPGTFLLR ELSAVTFPDIIR	0.24	0.56	Down
P14246	Solute carrier family 2, facilitated glucose transporter member 2	*Slc2a2*	HVLGVPLDDR VSVIQLFTDANYR	0.67	0.74	Down
P16858	Glyceraldehyde-3-phosphate dehydrogenase	*Gapdh*	IVSNASCTTNCLAPLAK LISWYDNEYGYSNR	0.58	0.73	Down
P14094	Sodium/potassium-transporting ATPase subunit beta-1	*Atp1b1*	VAPPGLTQIPQIQK YNPNVLPVQCTGK	0.50	0.69	Down
Q9R0H0	Peroxisomal acyl-coenzyme A oxidase 1	*Acox1*	TQEFILNSPTVTSIK AFTTWTANAGIEECR	0.44	0.68	Down
Pnlip	Pancreatic triacylglycerol lipase	*Pnlip*	TTYTQATQNVR ITGLDPAEPYFQGTPEEVR	0.05	0.25	Down
Q8CGA3	Large neutral amino acids transporter small subunit 4	*Slc43a2*	FSWLGFDHK	0.42	0.67	Down
P14483	H-2 class II histocompatibility antigen, A beta chain	*H2-Ab1*	AELDTVCR TEALNHHNTLVCSVTDFYPAK	0.41	0.65	Down
P36371	Antigen peptide transporter 2	*Tap2*	VEFQDVSFSYPR LVEHDQLR	0.31	0.61	Down
O70570	Polymeric immunoglobulin receptor	*Pigr*	NVDLQVLAPEPELLYK GVTGGSVAIACPYNPK	0.34	0.54	Down
Q9R233	Tapasin	*Tapbp*	VYHSSLPASGR ATAASLTIPR	0.20	0.48	Down
Q64374	Regucalcin	*Rgn*	TTSCCFGGK DGLNAEGLLR	3.21	2.93	Up
P03958	Adenosine deaminase	*Ada*	ANYSLNTDDPLIFK LNINAAK	1.81	1.98	Up
Q9DCG6	Phenazine biosynthesis-like domain-containing protein 1	*Pbld1*	LQPTDSFTQSSR GEPGGQTAPYDFYSR	2.83	2.13	Up
Q5BKQ4	Inactive pancreatic lipase-related protein 1	*Pnliprp1*	GSQTTYTQAANNVR NALSQIVDIDGIWSGTR	16.17	4.74	Up

## Discussion

The small intestine of mammals undergoes a process of intestinal remodeling during lactation to ensure that the intestine can adapt to the transition from milk to a solid diet (24). Wen L *et al.* and Choi J *et al.* reported that T_3_ and TRα have indispensable roles during the intestinal remodeling of amphibian metamorphosis (25, 26). *Thra* ^-/-^ and *Thra* ^-/-^ *Thrb* ^-/-^ (TR double knockout) mutations in *Xenopus tropicalis* were shown to result in reduced larval epithelial cell apoptosis and reduced adult stem cell formation/proliferation during metamorphosis (27, 28). Similarly, cell proliferation in the crypt was found to be reduced in both *Thra* knockout mice and Pax8^-/-^ mice (19, 20, 29). Recently, Yunbo Shi *et al.* reported that cell proliferation in the crypt and apoptosis on the villus were reduced in the adult intestine of *Thra* ^PV/+^ mice, and they speculated that the decreased cell proliferation in the crypt could lead to less epithelial cell death on the villus (1, 30).

Consistent with these findings, our intestinal proteomics analysis and GO analysis revealed a significant enrichment for the GO term developmental process, while the caspase-like domain was markedly enriched in the protein domain cluster analysis. Casp3, Casp7, and Ripk3, which are involved in the positive regulation of apoptotic processes (31–33), were downregulated. Ada, which inhibits epithelial cell apoptosis at the villous tips (34), was upregulated. Mki67 is a known marker of cell proliferation, and *Mki67* was proven to be a T_3_ positively regulated gene (35–37). Stat1, which is an important transcription factor, is involved in the regulation of cell proliferation (38). Both Mki67 and Stat1 were downregulated in the Hom mice, whereas Rgn, which negatively regulates cell proliferation, was upregulated (39). We speculate that these factors associated with cell proliferation and apoptosis could explain the dysplasia of the villi and the crypts of *Thra* ^E403X/E403X^ mice, but the underlying mechanism needs further investigation.

The small intestine is an important place for the digestion and absorption of nutrients, such as sugars, lipids, and proteins. A previous study reported that sucrase, lactase, and aminopeptidase enzymatic activities were decreased in purified the brush border membrane of the intestine of *Thra* ^-/-^ mice (24). Decreased lactase activity was also observed in the intestine of Pax8^-/-^ mice (29). These results suggest that TRα mutations or thyroid hormone deficiency could affect the metabolism of proteins and carbohydrates.

In the present study, several processes related to nutrient metabolism, such as fatty acid degradation (mmu00071), fatty acid metabolism (mmu0121), fat digestion and absorption (mmu04975), starch and sucrose metabolism (mmu00500), and protein digestion and absorption (mmu04974), were found to be enriched. Glyceraldehyde-3-phosphate dehydrogenase (Gapdh), which is negatively regulated by T_3_, was downregulated in the homozygous mice and plays a role in glycolysis (40, 41). Glucose transporter type 2 (Glut-2), which is mainly expressed in the intestine and participates in the transcellular transport of glucose in the intestine, was downregulated. It has also been reported that Glut-2 plays an important role in maintaining glucose homeostasis and the development of the pancreas (42). Sodium/potassium-transporting ATPase subunit beta-1(Atp1b1) is a sodium and potassium pump that provides a concentration gradient for the glucose transporters to absorb glucose from the intestinal lumen into the blood (43, 44). Its downregulation may affect the absorption of glucose in the intestine. For lipid metabolism, the triglyceride lipase inhibitor Pnliprp1 was significantly upregulated, suggesting a decreased efficiency of triglyceride digestion and absorption in the *Thra* ^E403X/E403X^ mice (45). Furthermore, Acox1 and Pnlip, which are associated with long fatty acid catabolism and cholesterol absorption (46, 47), were downregulated in the *Thra* ^E403X/E403X^ mice. For amino acid metabolism, the large neutral amino acid transporter small subunit 4 (Lat4) was significantly downregulated. Lat4 is mainly present in the crypts and plays an important role in amino acid (AA) transport across the cellular barrier in intestinal development (48–50). We speculate that these proteins associated with the absorption of nutrients could be associated with the impaired postnatal development of the *Thra* ^E403X/E403X^ mice.

The intestine is the largest lymphoid tissue in the body. A striking feature of the intestinal innate immune system is its ability to generate large amounts of noninflammatory immunoglobulin A (IgA) antibodies, which serve as the first line of host defense against intestinal pathogens (51). Our proteomic results showed that most of the DEPs related to antigen processing and presentation and the intestinal immune network for IgA production pathways were downregulated. Some representative proteins were quantified and validated by PRM. H2-Ab1 plays an important role in the presentation of MHC class II molecular antigens (52–54). Tap2 is a transporter associated with antigen processing (TAP) and acts as a molecular scaffold essential for peptide-MHC class I assembly and antigen presentation in complex with Tap1. Either Tap2 mutation or Tap2 deficiency (Tap2^-/-^) shows severely reduced expression of human lymphocyte antigen class I proteins on the cell surface, which affects the process by which antigen-presenting cells present antigens to CD8 T and NK cells (55, 56). Tapbp ensures the proper assembly of MHC class I molecules by interacting with TAP, and its function is conserved in both human and mouse cells (57). Pigr binds polymeric IgA at the basolateral surface of epithelial cells and is then transported across the cell to be secreted at the apical surface. Secreted IgA promotes immune exclusion by entrapping dietary antigens and microorganisms in the mucus and functions as a neutralizer of toxins and pathogenic microbes (58). The reduced levels of proteins related to intestinal IgA production and antigen presentation process suggest that the innate and adaptive immune barriers in the intestine could be impaired in *Thra* ^E403X/E403X^ mice.

In conclusion, our intestinal proteomic data indicate that TRα1 may play an important role in intestinal development. Except for the association with cell proliferation and apoptosis along the crypt–villus axis, which have been reported by previous studies, our intestinal proteomic results provide promising candidates for future studies as they suggest novel mechanisms by which TRα1 may influence intestinal development, such as the transport of intestinal nutrients and the establishment of innate and adaptive immune barriers of the intestine.

## Data Availability Statement

The datasets presented in this study can be found in online repositories. The names of the repository/repositories and accession number(s) can be found in the article/supplementary material.

## Ethics Statement

The animal study was reviewed and approved by the animal experimentation ethics committee of the China Medical University. Written informed consent was obtained from the owners for the participation of their animals in this study.

## Author Contributions

YX contributed to methodology and validation. DZ contributed to writing—original draft and review and editing. YL contributed to software and resources. ZS contributed to investigation and resources. XT contributed to formal analysis and validation. WT contributed to supervision and resources. All authors contributed to the article and approved the submitted version.

## Funding

This study was supported by Chinese National Natural Science Foundation grants 81970681 and 81570711 to XT.

## Conflict of Interest

The authors declare that the research was conducted in the absence of any commercial or financial relationships that could be construed as a potential conflict of interest.

## Publisher’s Note

All claims expressed in this article are solely those of the authors and do not necessarily represent those of their affiliated organizations, or those of the publisher, the editors and the reviewers. Any product that may be evaluated in this article, or claim that may be made by its manufacturer, is not guaranteed or endorsed by the publisher.
